# Pilot Randomized trial of Fibrinogen in Trauma Haemorrhage (PRooF-iTH): study protocol for a randomized controlled trial

**DOI:** 10.1186/s13063-016-1439-5

**Published:** 2016-07-19

**Authors:** Jacob Steinmetz, Anne Marie Sørensen, Hanne Hee Henriksen, Theis Lange, Claus Falck Larsen, Pär I. Johansson, Jakob Stensballe

**Affiliations:** Department of Anaesthesia and Trauma Centre, Centre for Head and Orthopaedics, Rigshospitalet, Copenhagen University Hospital, Blegdamsvej 9, 2100 Copenhagen, Denmark; Section for Transfusion Medicine, Capital Region Blood Bank Rigshospitalet, Copenhagen University Hospital, Blegdamsvej 9, 2100 Copenhagen, Denmark; Section of Biostatistics, University of Copenhagen, Oester Farimagsgade 5, 1014 Copenhagen, Denmark; Trauma Centre, Centre for Head and Orthopaedics, Rigshospitalet, Copenhagen University Hospital, Blegdamsvej 9, 2100 Copenhagen, Denmark; Department of Anaesthesia, Centre for Head and Orthopaedics and Section for Transfusion Medicine, Capital Region Blood Bank, Rigshospitalet, Copenhagen University Hospital, Blegdamsvej 9, 2100 Copenhagen, Denmark

**Keywords:** Trauma, Haemorrhage, Fibrinogen, Haemostatic Resuscitation, Thrombelastography

## Abstract

**Background:**

Haemorrhage remains a leading cause of morbidity and mortality in trauma patients. Fibrinogen is an essential endogenous component of haemostasis and the plasma level is associated with bleeding, transfusion and outcome. Fibrinogen concentrate is widely used to correct acquired hypofibrinogenaemia, recommended by several international guidelines for the treatment of trauma patients, but evidence is lacking regarding the treatment safety and efficacy.

We aim to assess the efficacy and safety of an immediate pre-emptive first-line treatment with fibrinogen concentrate in patients with trauma haemorrhage in need of haemostatic resuscitation.

**Methods/Design:**

This is a single-centre, randomized (1:1, active:placebo), placebo-controlled, double-blinded, investigator-initiated phase II trial. The trial population consists of 40 adult patients (>18 years) with traumatic, critical bleeding admitted to the Level 1 Trauma Centre at Rigshospitalet in Copenhagen, with immediate need for blood transfusion on arrival and an expected need for haemostatic resuscitation with multiple transfusions during the initial resuscitation. Patients will receive either pre-emptive administration of a bolus dose of 60–70 mg/kg fibrinogen concentrate (Riastap®) or placebo 0.9 % saline in equal volume to active treatment, both given as intravenous infusion blinded for the person administering the infusion.

The primary end point is the change in thrombelastograph (TEG®) functional fibrinogen maximum amplitude in millimetres at 15 min after the intervention. The follow-up period on safety events and mortality will be until day 30.

To detect a difference in the change from baseline to the 15-minute post-randomization measurement of 6–8 mm in TEG® functional fibrinogen maximum amplitude with a power of 0.90 and alpha of 0.05, we require 19 patients in each group. We have chosen to include 40 patients, 20 evaluable patients in each randomization group in case of attrition, in the present trial.

**Discussion:**

Patients considered to be included in the trial will temporarily have a compromised consciousness because of the acute, critical bleeding related to trauma, so scientific guardians will co-sign the informed consent form. Next of kin and the patients’ general practitioner or the patients will co-sign as soon as possible.

This trial will test whether immediate pre-emptive fibrinogen concentrate administered to adult trauma patients as first-line treatment of trauma haemorrhage will increase the clot strength as evaluated by thrombelastography, transfusion requirements and survival in patients receiving haemostatic resuscitation according to current standard of care.

**Trial registration:**

EudraCT no. 2014-003978-16 (22/1 2015); ClinicalTrials.gov: NCT02344069. Registered on 14 January 2015. Trial protocol version 4.2 (23-12-2014).

## Background

Haemorrhage remains a leading cause of morbidity and mortality in trauma patients [1]. Fibrinogen is an essential endogenous component of haemostasis [2] and the plasma level is associated to bleeding, transfusion and outcome [3]. Fibrinogen concentrate is widely used to correct acquired hypofibrinogenaemia, recommended by several guidelines [4, 5], but evidence is lacking regarding the treatment efficacy [6, 7].

Several trauma resuscitation strategies are currently being employed and investigated internationally [8]. The concept of haemostatic resuscitation is widely used in which early pre-emptive balanced transfusion therapy with red blood cells (RBC): fresh frozen plasma (FFP): platelets (PLT) are driven according to fixed ratios aiming at 1:1:1. The best evidence for this concept comes from Holcomb and colleagues, showing a beneficial effect of high ratios on haemostasis and short-term mortality due to exsanguination [9]. The Copenhagen Concept, Haemostatic Control Resuscitation (HCR), implemented in 2004, is a further adaptation of haemostatic resuscitation comprising of balanced transfusion therapy with transfusion packages equal to a ratio 1:1:1 in the early phase of massive bleeding, and then adjusting the therapy according to viscoelastic haemostatic assay-based algorithms allowing for goal-directed treatment of coagulopathy [1, 10]. The standard of trauma care in our facility is based on Advanced Trauma Life Support (ATLS) with strong focus on haemorrhage control, and early whole-body computed tomography (CT) if the patient is stable. Tranexamic acid is standard of both pre- and intrahospital care in Denmark according to the CRASH-2 trial [11]. HCR is associated with improved survival in observational studies, but still, in most resuscitation concepts, including HCR, a low level of fibrinogen is associated with coagulopathy, low clot strength and poor outcome [1, 3].

Fibrinogen is a key element not only in secondary haemostasis, where it is cleaved by thrombin to form fibrin, but also in primary haemostasis by facilitating platelet aggregation and adhesion [12]. Fibrinogen levels average 2–4 g/l in healthy individuals and have been shown to be the element of the coagulation system that first reaches low levels during haemorrhage, at least if volume replacement is based on RBC and fluids alone [13], and in conditions with hyperfibrinolysis [14]. The “critical level” at which acquired fibrinogen deficiency causes a decrease in haemostatic competence is, however, debatable. Studies in patients with congenital fibrinogen deficiencies indicate that only levels below 1 g/l cause an increase in bleeding during surgery [6], and this level has generally been adopted as a target for substitution in bleeding patients with acquired deficiencies also [6, 15]. A level as high as 1.5–2 g/l is now being suggested as the target for substitution since decreased levels have been associated with worse outcomes in bleeding patients [3–5, 16, 17]. In regard to the use of fibrinogen concentrate in bleeding trauma patients, no data from prospective randomized controlled trials (RCT) have been published. However, there are another ongoing study (NCT01475344), and the FiiRST study (NCT02203968) that is completed, but with no results published yet. The CRYOSTAT pilot trial showed that cryoprecipitate supplementation containing fibrinogen in trauma is feasible and that a definitive trial is warranted to see the effect on safety, transfusions, morbidity and mortality [18]. However, cryoprecipitate is more than fibrinogen substitution since all coagulation factors are in there and it may not be comparable to fibrinogen concentrate primarily containing fibrinogen [19]. Outside trauma, the evidence for use of fibrinogen is also very scarce and most studies are with high risk of bias [15]. Moreover, in the latest RCT on severe postpartum haemorrhage no evidence to support the preemptive use of 2 g fibrinogen concentrate was found, however no concern related to safety and thromboembolic complications in particular appeared [20].

The objective of this trial is to assess the efficacy and safety of an immediate pre-emptive first-line treatment with fibrinogen concentrate in patients with trauma haemorrhage in need of haemostatic resuscitation. We hypothesize that immediate pre-emptive fibrinogen concentrate administered to adult trauma patients as first-line treatment of traumatic haemorrhage will increase the clot strength as evaluated by thrombelastography (TEG®).

## Methods/design

This is a single-centre randomized, double-blinded, controlled, investigator-initiated, pilot phase II trial of 40 haemorrhaging trauma patients admitted to the Level 1 Trauma Centre in Copenhagen, Denmark randomized to administration of fibrinogen concentrate (Riastap®, CSL Behring Scandinavia, Danderyd, Sweden), as compared to placebo (saline), as immediate, pre-emptive first-line treatment of trauma haemorrhage, when haemostatic resuscitation is needed.

### Patients

The study will include 40 adult (≥18 years) trauma patients received directly from the scene of the accident, where an order of at least one blood component transfusion within the first hour of arrival is initiated, and the patient is predicted to need transfusion package therapy during the first 2 hours (activated massive transfusion protocol) [10]. Detailed inclusion and exclusion criteria are listed in Table 1. A written informed consent will be obtained from patients or scientific guardians (independent physicians and/or next of kin).

**Table 1:** Selection criteria

Inclusion criteria
• Trauma patient received directly from the scene of the accident AND • Age ≥18 years AND • Initiated order of transfusion of at least one blood component within the first hour of arrival AND • Predicted to need transfusion package therapy during the initial resuscitation (first 2 hours) AND • Consent obtainable from patient or scientific guardians (independent physicians and/or next of kin)
Exclusion criteria
Patients are not eligible for inclusion in this trial if they fulfill one or more of the following criteria: • Duration of >2 hours from time of accident to arrival at trauma centre OR • Known anticoagulant treatment (vitamin K antagonist, dabigatran, rivaroxiban, apixaban) OR • Severe isolated traumatic brain injury OR • Moribund patient with devastating injuries and expected to die within 1 hour of admission OR • Withdrawal from active therapy OR • Previously, within 30 days, included in a randomized trial, if known at the time of enrolment OR • Known body weight <55 kg OR • Any blood product prior to inclusion

### Randomization

The randomization is done in blocks of six, and the randomization sequence and envelopes are generated and validated by two persons that are otherwise not involved in the trial. Randomization was performed using Microsoft Excel software (Microsoft Corp., Redmond, WA, USA). Treatments (fibrinogen concentrate - Riastap® or placebo/saline) were arranged in two groups of 20 patients in a single column in a systematic order. Each block of six patients included three fibrinogen concentrate - Riastap® and three placebo/saline patients. A second column was created for random numbers and the treatments column was then sorted by these random numbers [21].

Two identical sets of envelopes are generated - one set for randomization of patients at the Trauma Centre, and an “emergency” set for code breaking if necessary. Written and signed documentation for the double-controlled generation of randomization envelopes is kept at the Blood Bank. The randomization sequence is kept secret for the investigators by use of successively numbered, sealed, and opaque envelopes. Each patient is given a unique randomization number.

Emergency department nurses in the Trauma Centre, not involved in treatment of the specific patient, will perform 24-hour on-site randomization by envelope-opening to allow for immediate allocation to either receiving fibrinogen concentrate (intervention) or saline (placebo). A written instruction for the randomization procedure is kept with the envelopes.

A randomization list is held by the sponsor, which will be available to the investigator after the completion of the trial. Emergency code-breaking envelopes are available in the Blood Bank where they can be opened if decided by the sponsor.

### Interventions

The treating physician must have evaluated the patient’s eligibility and approved the patient’s enrolment in the trial prior to administration of the trial drug/placebo. All patients will be treated according to normal standard of care for haemorrhaging trauma patients.

**Fig. 1:**
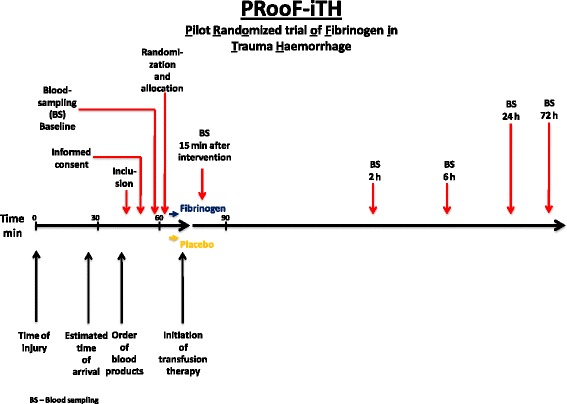
Trial flow diagram

The trial/placebo drug is administered as an intravenous bolus dose as early as possible during the initial resuscitation (Fig. 1). During the infusion the patient will be observed especially with regard to potential signs of thrombotic and allergic complications.

#### Experimental arm

The active treatment (n = 20 patients) consists of intravenous injection of fibrinogen concentrate (Riastap®) of 60–70 mg/kg (dose of 4 g for patients with body weight 55–69 kg, 5 g for 70–85 kg or 6 g for >85 kg) as a bolus dose when haemostatic resuscitation is deemed necessary by the clinician. Fibrinogen is administered as an immediate single intravenous injection, according to Solomon et al. [22, 23]. The aim is to give the intervention <1 hour of arrival, and the intervention should, when possible, be given prior to blood products.

#### Control arm

Patients in the placebo group (n = 20 patients) will receive 0.9 % saline infusions in equal volume to active treatment and will be treated exactly as active patients. The volume of placebo administered is equal to the volume of active drug administered, again as a bolus dose when haemostatic resuscitation is deemed necessary by the clinician.

The content (Riastap® or placebo) will be provided in opaque syringes and infusion sets (yellow-coloured) to disguise the content of the allocation to the treatment team.

## Trial end points

The primary end point is the change in thrombelastograph TEG® functional fibrinogen (FF) maximum amplitude (MA) in millimetres at 15 min after the intervention (Table 2). Secondary end points are shown in Table 2 and include the clinically important 24-hour, and 30-day mortality.

**Table 2:** Trial end points

Primary end point
Thrombelastograph (TEG®) functional fibrinogen (FF) maximum amplitude (MA) in millimetres at 15 min post intervention
Secondary end points
• TEG® FF MA in millimetres at 2, 6, 24 and 72 hours post intervention • TEG® MA in millimetres at 15 min, 2, 6, 24 and 72 hours post intervention • Transfusion requirements (red blood cells (RBC) or fresh frozen plasma (FFP) or platelets (PLT)) at 2, 6, 24, 72 hours and in total at day 30 • Total use of haemostatic therapy (i.e. use of coagulation factor concentrates and tranexamic acid) in the first 24 and 72 hours, omitted from this is active treatment (intervention) • Time to intervention or placebo • Time to FFP and PLT transfusion • Percentage of patients receiving intervention or placebo <1 hour of arrival • Time to surgical control of bleeding as noted by the surgeon • Severe adverse reactions at day 30, defined as symptomatic thromboembolism at day 30 and anaphylaxis at day 30 • 24-hour and 30-day mortality

Follow-up period on safety events and mortality will be until day 30.

During the trial blood samples will be taken at different time points (Table 3). Patients will be actively assessed for the first 72 hours from admission. During the extended follow-up period at day 30, contact will be made with the patients and/or department to follow up on safety events and establish potential mortality (Table 3). Data collection forms can be obtained from the authors.

**Table 3:** Observations and blood sampling

		Time point					
Day	Day 1	Day 1	Day 1	Day 1	Day 2	Day 4	Day 30 (follow-up)
*Hours*	*0 hrs* baseline (before infusion)	*15 min*	*2 hrs*	*6 hrs*	*24 hrs*	*72 hrs*	
Informed consent	X						
Demographics/medical history					X	X	X
Weight	X						
Clinical parameters^a^	X	X	X	X	X	X	
Co-medication^b^, incl. fluids/blood	X	X	X	X	X	X	
Haematology/biochemistry	X	X	X	X	X	X	
Thrombelastography	X	X	X	X	X	X	
Drug/placebo administration	X						
Serious adverse events							X
Mortality					X		X

Time points are calculated from infusion of study drug

^a^Clinical parameters including lactate, blood pressure and pulse

^b^Only concomitant medication affecting the haemostasis are registered

## Statistical analysis

All efficacy analyses will be done according to the intention-to-treat principle comprising all patients who receive intervention (active or placebo), even if not completed. This includes all randomized patients fulfilling inclusion criteria and not meeting exclusion criteria. The per protocol population will be a subset of the intention-to-treat population. It includes patients who have received the intervention (active or placebo), but excluding the following patients: patients receiving blood products between time of inclusion and intervention or patients receiving haemostatics such as fibrinogen concentrate, prothrombin complex concentrate or recombinant factor VIIa after the administration of intervention (active or placebo).

Statistical analyses will be performed by a statistician blinded to the allocated treatment prior to the breaking of the randomization code. All outcomes will be reported according to analyses described above. Descriptive statistics will be calculated for all end points. All summary statistics of continuous variables will include: n, mean with standard deviation, or median with interquartile ranges. All summary statistics of frequency tables will include n, % and N, where N is the total number of patients recorded values in the corresponding group.

The change in TEG® FF MA from baseline to the 15-min measurement will be analysed with ANCOVA. The analysis will be adjusted for baseline TEG® FF MA to increase statistical power. The assumption of normality of the residuals will be assessed using QQ-plots and, if a satisfactory fit is not obtained, we will first attempt to conduct the analysis using log-transforms. If the fit is still not adequate, we will employ the Mann-Whitney test to the change values. The primary null hypothesis is that there is no difference between the fibrinogen concentrate arm and the placebo arm.

The secondary end points (number 1–4; Table 2) will be analysed using mixed models, adjusting for baseline values. The models will include an interaction term between treatment group and time. If an overall significant treatment effect is found post hoc pair-wise comparisons will be employed. The assumption of normality of the residuals will be assessed using QQ-plots and, if a satisfactory fit is not obtained, we will first attempt to conduct the analysis using log-transforms. If the fit is still not adequate, we will employ the Mann-Whitney test to the measured changes from baseline to each of the measurement times; i.e. there will be as many Mann-Whitney tests as there are measurement times.

The secondary outcomes (number 5–7; Table 2) will be analysed using Cox models as these are time-to-event outcomes with potential competing risks. The assumption of proportional hazards will be assessed using Schoenfeld residuals. Secondary outcomes (number 7, 9, 10; Table 2) are all categorical and will be analysed using Fisher’s exact test and effects quantified using frequency tables and risk ratios.

### Missing data

If for any of the analyses mentioned above there are less than 95 % of the observations which are complete, we will employ multiple imputation techniques for that analysis [24]. The multiple imputation approach will be based on chained equations. If more than 95 % of the observations are complete, we will only employ complete case analysis.

The power calculation is based on local data finding of a TEG® FF MA mean 13.6 mm (SD ± 5.6) on arrival in trauma patients not surviving for 30 days. Administering 60–70 mg/kg fibrinogen concentrate will result in an approximate increase of 6–8 mm in TEG FF MA according to Tanaka and colleagues, which corresponds to increasing the fibrinogen level with approximately 1 g/l and to a level of above 2.3 g/l [25]. To detect the above difference with a power of 0.90 (1-β) and alpha of 0.05 requires n = 19 patients in each group. We have chosen to include 40 patients, 20 evaluable patients in each randomization group in case of attrition, in the present trial. Patients who drop out or are withdrawn for any reason before hour 24 will be replaced.

No interim analysis is planned. The actual statistical power will be a bit higher as the primary end point is a change value and we adjust the analysis for baseline values. However, the exact size of this additional power cannot be readily assessed as we do not have reliable information about the intra-person correlation of TEG® FF MA.

### Patient withdrawal

Participation in the trial is strictly voluntary. Patients, relatives or scientific guardians are free to discontinue the trial at any time without giving their reason(s).

A patient must be withdrawn from the trial treatment in the event of any of the following:

1. Withdrawal of consent, or
2. Due to serious adverse reactions (anaphylaxis, or suspected unexpected serious adverse reactions), which are clinically relevant and affect the patient’s safety, and discontinuation is considered necessary by the trial investigators (stopping rule). In case a stopping rule emerges, the sponsor in concert with the investigator/treating physician decides whether code breaking should be performed. Emergency code-breaking envelopes are stored at the Blood Bank and are opened at the initiative of the sponsor. It is documented in the case report form (CRF), if the code is broken.

All patients who withdraw from the trial for any reason and at any time should have an end-of-trial examination. Patients will be examined for any status changes that require further follow-up. All withdrawn patients will be followed up as the remaining patients in the trial. If consent is withdrawn, the person making the withdrawal will be asked for permission to follow up for 30 days after randomization.

## Discussion

A low level of fibrinogen is associated with coagulopathy, low clot strength and poor outcome, and fibrinogen concentrate is currently being used at various facilities at the discretion of the physician on a case-by-case basis, often goal-directed according to viscoelastic haemostatic assays such as thrombelastography (TEG®) or thrombelastometry (ROTEM®)[1, 3]. The Pilot Randomized trial of Fibrinogen in Trauma Haemorrhage (PRooF-iTH) is the first randomized controlled trial of fibrinogen in trauma. Furthermore, the first trial to address whether first-line, pre-emptive fibrinogen concentrate treatment will increase the clot strength, and influence transfusion requirements and survival in adult trauma patients in need of haemostatic resuscitation. Included patients will have critical or massive bleeding in need of activation of our massive transfusion protocol.

When administering a pro-haemostatic drug such as fibrinogen concentrate, there is a possible risk of thromboembolic events. In seven randomized controlled trials on the use of fibrinogen concentrate as a pro-haemostatic drug, six of these were in cardiac surgery carrying an inevitable increased thromboembolic risk, and only one trial has reported two cases of thromboembolic events [26]. However, in a 22-year pharmaco-surveillance programme with more than 1,000,000 g of fibrinogen concentrate administered, only nine cases of thrombosis possibly related to the treatment have been reported [27]. Furthermore, in a recent randomized controlled trial using 2 g fibrinogen substitution or placebo in 249 patients with severe postpartum haemorrhage done by our group, we saw no thromboembolic events and no serious adverse events or reactions [28]. In conclusion, the risk of thromboembolic events seems negligible when fibrinogen is used in a relevant population, with potential important benefits.

The dose of fibrinogen concentrate in the present trial is 60–70 mg/kg and was chosen based on the reported findings of low concentration of fibrinogen in actively haemorrhaging trauma patients [29, 30] and it is estimated that the chosen dose will increase the functional fibrinogen maximal amplitude by 6–8 mm resulting in significantly higher overall clot strength [25]. Importantly, no clinical or preclinical data indicate that administration of fibrinogen concentrate to patients having a normal fibrinogen leads to a hypercoagulable or pro-thrombotic state [20].

In this trial we wish to include pregnant women and find that advantages outweigh possible risks. These trauma patients are bleeding critically and therefore receive many blood transfusions, and 20 % are expected to die from their injuries. It will not be possible to investigate for pregnancy prior to administration of trial medication, as this will delay immediate and life-saving treatments. The active substance in Riastap® is of human origin and is eliminated in the same manner as the native protein. Harmful or toxic effects in relation to reproduction or foetal toxicity are not expected, and fibrinogen concentrate has safely been used for postpartum haemorrhage in more than 100 women without any serious adverse reactions or events even though this is not the same as being pregnant. Despite the several measures taken to screen for and inactivate viruses and bacteria, the risk of non-enveloped viruses such as parvovirus B19, which may be serious for pregnant women, cannot be completely excluded [20]. Overall, we strongly believe that the advantages of including pregnant women outweigh possible risks and adverse reactions. Based on this, the inclusion of pregnant women was approved by the Danish health authorities.

The future of trauma resuscitations warrants further investigation. Currently, the best evidence from randomized controlled trials supports haemostatic resuscitation aiming at delivering blood products in a ratio 1:1:1 of red blood cells, plasma and platelets during resuscitation of bleeding. These results comes from the Pragmatic, Randomized Optimal Platelets and Plasma Ratios trial (PROPPR) [9], showing a reduction in numbers of patients exsanguinating, a lower 6-hour mortality and tendency towards a better 24-hour mortality. The PRooF-iTH will test whether immediate pre-emptive fibrinogen concentrate administered to adult trauma patients as first-line treatment of trauma haemorrhage will increase the clot strength as evaluated by thrombelastography (TEG®), transfusion requirements and survival, as compared to placebo in patients receiving standard of care resuscitation. In brief, the standard of trauma care in Copenhagen is performed according to the ATLS principles and resuscitation is done according to the Damage Control Resuscitation principles [31], the PROPPR trial [9] and the developed Copenhagen Concept [10] where we limit the use of fluids and initiate a 1:1:1 ratio-driven transfusion therapy of RBCs, FFP, and platelets during the initial phase of massive bleeding. Viscoelastic haemostatic assay (VHA) is performed on arrival and repeated accordingly, allowing for an early shift towards VHA-guided therapy using blood products, pro-haemostatics and coagulation factor concentrates subsequently. Simultaneously, tranexamic acid is administered according to the CRASH-2 trial [11], and efforts are made to correct and reverse augmenting factors of coagulopathy and shock [10].

## Trial status

The trial was started in February 2015, and participants are currently being recruited. The estimated trial completion will be in December 2016.

## Abbreviations

ATLS, Advanced Trauma Life Support; FF, functional fibrinogen; FFP, fresh frozen plasma; HCR, Haemostatic Control Resuscitation; MA, maximum amplitude; PLT, platelets; PRooF-iTH, Pilot Randomized trial of Fibrinogen in Trauma Haemorrhage trial; RBC, red blood cells; RCT, randomized controlled trial; TEG®, thrombelastograph; VHA, viscoelastic haemostatic assay

## References

[1] Johansson PI, Sorensen AM, Larsen CF, Windeløv NA, Stensballe J, Perner A (2013). Low hemorrhage-related mortality in trauma patients in a Level I trauma center employing transfusion packages and early thromboelastography-directed hemostatic resuscitation with plasma and platelets. Transfusion.

[2] Hoffman M, Cichon LJ (2013). Practical coagulation for the blood banker. Transfusion.

[3] Rourke C, Curry N, Khan S, Taylor R, Raza I, Davenport R (2012). Fibrinogen levels during trauma hemorrhage, response to replacement therapy and association with patient outcomes. J Thromb Haemost.

[4] Spahn DR, Bouillon B, Cerny V, Coats TJ, Duranteau J, Fernández-Mondéjar E (2013). Management of bleeding and coagulopathy following major trauma: an updated European guideline. Crit Care.

[5] Kozek-Langenecker SA, Afshari A, Albaladejo P, Santullano CA, De Robertis E, Filipescu DC (2013). Management of severe perioperative bleeding: guidelines from the European Society of Anaesthesiology. Eur J Anaesthesiol.

[6] Wikkelso A, Lunde J, Johansen M, Stensballe J, Wetterslev J, Møller AM (2013). Fibrinogen concentrate in bleeding patients. Cochrane Database Syst Rev.

[7] Meyer MA, Ostrowski SR, Windelov NA, Johansson PI (2011). Fibrinogen concentrates for bleeding trauma patients: what is the evidence?. Vox Sang.

[8] Stensballe J, Ostrowski SR, Johansson PI (2014). Viscoelastic guidance of resuscitation. Curr Opin Anaesthesiol.

[9] Holcomb JB, Tilley BC, Baraniuk S, Fox EE, Wade CE, Podbielski JM (2015). Transfusion of plasma, platelets, and red blood cells in a 1:1:1 vs a 1:1:2 ratio and mortality in patients with severe trauma: the PROPPR randomized clinical trial. JAMA.

[10] Johansson PI, Stensballe J, Oliveri R, Wade CE, Ostrowski SR, Holcomb JB (2014). How I treat patients with massive hemorrhage. Blood.

[11] Shakur H, Roberts I, Bautista R, Caballero J, Coats T, CRASH-2 trial collaborators (2010). Effects of tranexamic acid on death, vascular occlusive events, and blood transfusion in trauma patients with significant haemorrhage (CRASH-2): a randomised, placebo-controlled trial. Lancet.

[12] Lang T, Johanning K, Metzler H, Piepenbrock S, Solomon C, Rahe-Meyer N (2009). The effects of fibrinogen levels on thromboelastometric variables in the presence of thrombocytopenia. Anesth Analg.

[13] Hiippala ST, Myllyla GJ, Vahtera EM (1995). Hemostatic factors and replacement of major blood loss with plasma-poor red cell concentrates. Anesth Analg.

[14] Cotton BA, Harvin JA, Kostousouv V, Minei KM, Radwan ZA, Schöchl H (2012). Hyperfibrinolysis at admission is an uncommon but highly lethal event associated with shock and prehospital fluid administration. J Trauma Acute Care Surg.

[15] Lunde J, Stensballe J, Wikkelso A, Johansen M, Afshari A (2014). Fibrinogen concentrate for bleeding - a systematic review. Acta Anaesthesiol Scand.

[16] Dzik WH, Blajchman MA, Fergusson D, Hameed M, Henry B, Kirkpatrick AW (2011). Clinical review: Canadian National Advisory Committee on Blood and Blood Products--Massive transfusion consensus conference 2011: report of the panel. Crit Care.

[17] Thomas D, Wee M, Clyburn P, Walker I, Brohi K, Association of Anaesthetists of Great Britain and Ireland (2010). Blood transfusion and the anaesthetist: management of massive haemorrhage. Anaesthesia.

[18] Curry N, Rourke C, Davenport R, Beer S, Pankhurst L, Deary A (2015). Early cryoprecipitate for major haemorrhage in trauma: a randomised controlled feasibility trial. Br J Anaesth.

[19] Jensen NH, Stensballe J, Afshari A (2016). Comparing efficacy and safety of fibrinogen concentrate to cryoprecipitate in bleeding patients: a systematic review. Acta Anaesthesiol Scand.

[20] Wikkelsø AJ, Edwards HM, Afshari A, Stensballe J, Langhoff-Roos J, Albrechtsen C, FIB-PPH trial group (2015). Pre-emptive treatment with fibrinogen concentrate for postpartum haemorrhage: randomized controlled trial. Br J Anaesth.

[21] Altman DG, Bland JM (1999). How to randomise. BMJ.

[22] Solomon C, Pichlmaier U, Schoechl H, Hagl C, Raymondos K, Scheinichen D (2010). Recovery of fibrinogen after administration of fibrinogen concentrate to patients with severe bleeding after cardiopulmonary bypass surgery. Br J Anaesth.

[23] Solomon C, Hagl C, Rahe-Meyer N (2013). Time course of haemostatic effects of fibrinogen concentrate administration in aortic surgery. Br J Anaesth.

[24] Carpenter JR, Kenward MG (2013). Multiple imputation and its application (Statistics in practice).

[25] Tanaka KA, Esper S, Bolliger D (2013). Perioperative factor concentrate therapy. Br J Anaesth.

[26] Karlsson M, Ternstrom L, Hyllner M, Baghaei F, Flinck A, Skrtic S (2009). Prophylactic fibrinogen infusion reduces bleeding after coronary artery bypass surgery. A prospective randomised pilot study. Thromb Haemost.

[27] Dickneite G, Pragst I, Joch C, Bergman GE (2009). Animal model and clinical evidence indicating low thrombogenic potential of fibrinogen concentrate (Haemocomplettan P). Blood Coagul Fibrinolysis.

[28] Innerhofer P, Westermann I, Tauber H, Breitkopf R, Fries D, Kastenberger T (2013). The exclusive use of coagulation factor concentrates enables reversal of coagulopathy and decreases transfusion rates in patients with major blunt trauma. Injury.

[29] Meyer AS, Meyer MA, Sorensen AM, Rasmussen LS, Hansen MB, Holcomb JB (2014). Thrombelastography and rotational thromboelastometry early amplitudes in 182 trauma patients with clinical suspicion of severe injury. J Trauma Acute Care Surg.

[30] Meyer MA, Ostrowski SR, Sørensen AM, Meyer AS, Holcomb JB, Wade CE (2015). Fibrinogen in trauma, an evaluation of thrombelastography and rotational thromboelastometry fibrinogen assays. J Surg Res.

[31] Holcomb JB, Jenkins D, Rhee P, Johannigman J, Mahoney P, Mehta S (2007). Damage control resuscitation: directly addressing the early coagulopathy of trauma. J Trauma.

